# Patterns of theta oscillation reflect the neural basis of individual differences in epistemic motivation

**DOI:** 10.1038/srep29245

**Published:** 2016-07-06

**Authors:** Patrick Mussel, Natalie Ulrich, John J. B. Allen, Roman Osinsky, Johannes Hewig

**Affiliations:** 1 Julius Maximilians University Würzburg, Department of Psychology I, Würzburg, Germany; 2 University of Arizona, Department of Psychology, Tucson, Arizona, USA; 3 University of Osnabrück, Department of Psychology, Osnabrück, Germany

## Abstract

Theta oscillations in the EEG have been shown to reflect ongoing cognitive processes related to mental effort. Here, we show that the pattern of theta oscillation in response to varying cognitive demands reflects stable individual differences in the personality trait epistemic motivation: Individuals with high levels of epistemic motivation recruit relatively more cognitive resources in response to situations possessing high, compared to low, cognitive demand; individuals with low levels do not show such a specific response. Our results provide direct evidence for the theory of the construct need for cognition and add to our understanding of the neural processes underlying theta oscillations. More generally, we provide an explanation how individual differences in personality traits might be represented on a neural level.

Cognitive achievements are the driving force for many important life outcomes, such as academic success^1^, job performance^2^, or innovations^3^, with a large positive impact for the individual and, in some cases, society as a whole. Besides cognitive abilities^4^, a major predictor for cognitive achievements is epistemic motivation^5^,^6^,^7^. Individual differences in epistemic motivation refer to the desire to develop and maintain a rich and accurate understanding of the world^8^. A prominent construct pertaining to the domain of epistemic motivation that has received considerable research attention is *need for cognition*^9^, defined as the enjoyment of and the tendency to engage in complex and cognitively challenging endeavors. According to an influential theory of this construct, individuals with high levels of need for cognition devote more cognitive effort to cognitive processing in complex, compared to simple problems, whereas individuals with low levels of need for cognition have been described as cognitive misers, with a lack of motivation to engage in cognitive challenges and a preference for simple tasks^9^. In the present study, we investigate the neural basis of this personality theory by relating need for cognition to theta oscillations in the EEG in response to varying levels of cognitive demands.

Theta oscillations are a potential indicator of cognitive effort. Power in the theta band has been shown to reflect ongoing cognitive processes related to a number of different cognitive tasks, especially involving working memory, executive control, and short-term memory load^10^,^11^,^12^,^13^,^14^,^15^. It is sensitive to endogenously generated motor responses and exogenously evoked percepts associated with novelty, conflict, punishment and errors and has thus been proposed to act to organize neural processes during decision points, therefore reflecting a need for cognitive control or for cognitive conflict detection and signaling^16^,^17^,^18^. Theta power has been found to reflect comprehensive functional brain states involved in encoding and retrieving information associated with temporal and hippocampal hemodynamic activation, widespread cingulate activation, frontal superior activation, and cerebellar activation^19^. Therefore, a prominent view is that power in the theta band reflects a binding process of widely distributed cortical assemblies or, more generally, mental effort^20^,^21^.

Here, we investigate whether individual response in theta power to varying levels of cognitive demands within a task may reflect individual differences in epistemic motivation. Specifically, we hypothesize that individuals with high epistemic motivation, as reflected in psychometric need for cognition, recruit relatively more cognitive resources in response to situations possessing high, compared to low, cognitive demands; individuals with low epistemic motivation should show an opposite pattern. Subsequently, we use this indicator to predict cognitive achievement and, thus, investigate whether the individual pattern of theta activity may serve as a neural trait of epistemic motivation.

## Results

**Figure 1:**
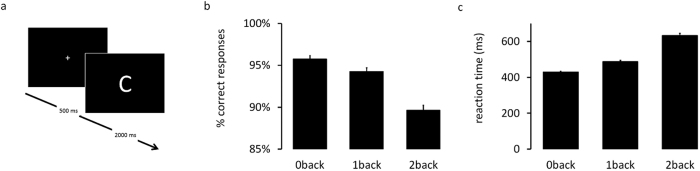
(**a**) Each trial of the n-back task consists of a fixation-cross and a capital letter. In the first block (condition 0-back), the letter “X” was the target. In the second block (condition 1-back), the target was the letter from the previous trial, and in the third block (condition 2-back), the letter presented two trials ago served as target. Participants were instructed to respond with a button press of their right index finger for targets, and with their left index finger for non-targets. There were 62 trials in each block (50% targets). (**b**) Performance (percentage of correct response) and (**c**) reaction times in the three conditions. Error bars indicate 95%-confidence intervals.

We used the n-back task^22^ to manipulate cognitive effort. Participants were instructed to monitor a sequence of stimuli (capital letters) and to respond whether a stimulus presented is the same as the one presented n trials previously, with n varying from 0 to 2 (see Fig. 1a). For n = 0, participants had to compare the letter of the present trial with a fixed letter (i.e. ”X”) whereas for n = 1 and n = 2, they had to compare it to the letter presented in the last or second to last trial. With increasing numbers of n, mental load increases with regards to updating and memorizing the target. Participants performed generally well on the task. On average, they responded correctly in 93% of the trials. Condition (i.e., 0-back vs. 1-back vs. 2-back) had a substantial influence on performance (F = 18.6, p ≤ 0.001). As cognitive load increased, performance decreased, with significantly less hits in 1-back, compared to 0-back (t = 2.4; p = 0.016) and significantly less hits in 2-back, compared to 1-back (t = 4.5; p < 0.001). Reaction time was on average 516 ms (SD = 230 ms) and increased with higher levels of cognitive demand (F = 46.2; p < 0.001; see Fig. 1b). All three conditions differed on p < 0.001 from each other.

### Neural processes

**Figure 2:**
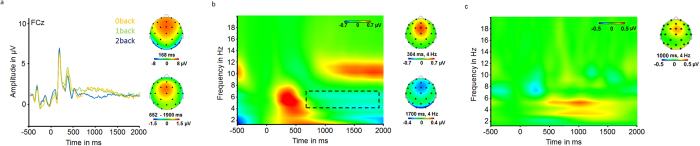
(**a**) Event-related responses, averaged across trials and separate for conditions (i.e. 0-back, 1-back, and 2-back), relatively to the onset of the stimulus. Topoplots represent data aggregated across the three conditions. (**b**) Results from time-frequency analysis revealed an early (0–650 ms), stimulus-driven neural reaction characterized by an increase of theta relative to the baseline, and a late (650–1900 ms) process which might reflect relaxation after the response, preparation of the upcoming trial and updating, memorizing and rehearsal of the target stimulus. Data in theta band (4–7 Hz) of the late process, see black rectangle, were extracted and averaged across channels Fz, FC1, FC2, FCz, Cz for further analyses. (**c**) Time-frequency difference plot to illustrate the stronger rise in late theta power from 0back to 2back for individuals high, compared to low in need for cognition. Computed as a double difference [2back_high_-0back_high_]-[2back_low_-0back_low_], where high and low refer to subgroups of individuals high and low in need for cognition, computed via median.

We recorded brain-related activity during the task via the EEG. Relative to the onset of the letter, averaged across trials and conditions, we found a large positive deflection 168 ms after stimulus onset with a fronto-central distribution (channels Fz, FC1, FC2, FCz, Cz; see Fig. 2a). A similar topography was found during the late time window between 650 and 1900 ms, which might signal ongoing activity with regards to the processing of the stimulus and preparation for the next trial.

We performed a Time-Frequency Analyses with a Complex Morlet Wavelet within frequencies ranging from 1 to 20 Hz to estimate fluctuations in power following the onset of the stimulus relative to a baseline interval (−150 to −50 ms). Across trials and conditions, time-frequency analysis revealed two separable processes during the 2-second interval between the presentation of the letter and the following fixation cross (Fig. 2b): First, an early process between 0 and 650 ms, characterized by an increase in theta (i.e. 4 to 7 Hz) and low levels of alpha (i.e. 8 to 13 Hz), which might be related to the evaluation of the stimulus and response preparation. Second, a late process between 650 and 1900 ms characterized by decrease in theta relative to baseline and an increase in alpha power. This second process might reflect relaxation after the response, preparation of the upcoming trial and updating, cognitive control, memorizing and rehearsing the target stimulus.

**Figure 3:**
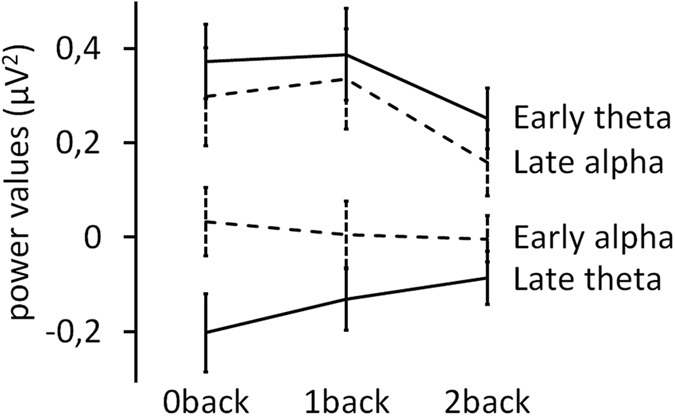
Alpha (8–13 Hz) and theta (4–7 Hz) power as a function of condition (0-back; 1-back; 2-back) for an early (0–650 ms) and a late (650–1900 ms) time window following the presentation of the stimulus. Error bars indicate 95%-confidence intervals.

We investigated the influence of cognitive demand possessed by the three levels of the n-back task on alpha (8–13 Hz) and theta (4–7 Hz) power for the two time windows. Generally, we found that cognitive effort shifted from early to late processes as cognitive demand increased. Specifically, we found a significant decrease in early theta activity in the 2-back condition, compared to the 1-back and 0-back condition (F = 6.8; p = 0.001). Additionally, late alpha power in the 8–13 Hz band decreased (F = 6.9; p = 0.001) and late theta power increased (F = 4.4; p = 0.012, see Fig. 3). Enhanced levels of cognitive effort have been associated with increased theta and reduced alpha activity^20^. Therefore, this pattern is likely a result of the increasing demands in maintaining and updating the stimulus in the 1-back, compared to the 0-back condition as well as in the 2-back, compared to the 1-back condition. Differences between these conditions are associated with relatively larger ongoing cognitive effort of executive functions, compared to early stimulus-related demands such as stimulus evaluation, decision-making and motor-response-related activity^22^. According to these results and based on theoretical considerations, we next focused on this latter process as it is less stimulus-driven and might, therefore, be more strongly related with top-down processes associated with varying levels of epistemic motivation.

### Latent growth curve modeling

**Figure 4:**
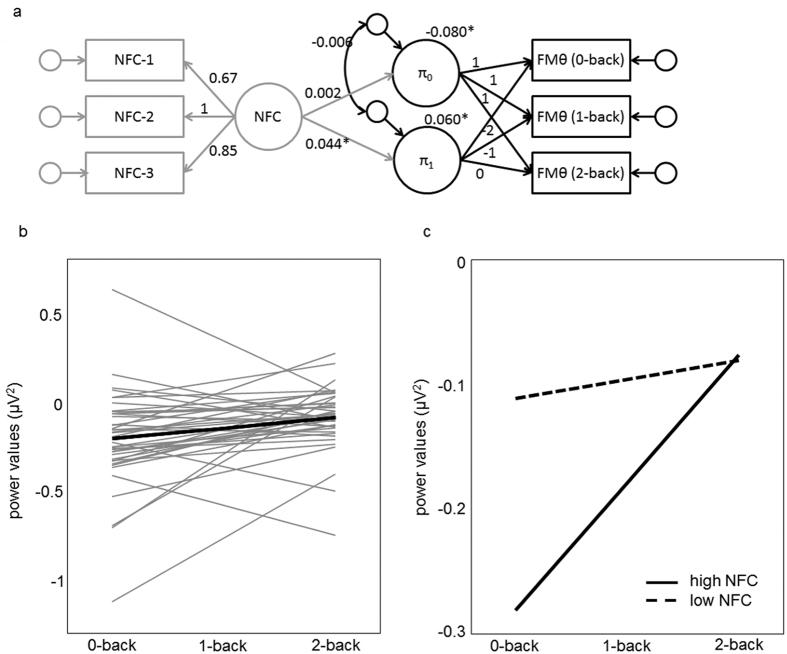
(**a**) Graphical depiction of the latent growth curve model with an intercept (π_0_) and a slope (π_1_) used to estimate patterns of responses in theta power (FMθ) between 650 and 1900 ms relative to the baseline as a function of cognitive load. In grey, slope and intercept are predicted by need for cognition (NFC). Values refer to unstandardized estimates from the latter model. (**b**) In grey, fitted ordinary least square linear regressions for the 42 participants are shown. In black, the level-2 trajectory is shown, indicating a mean increase in theta power as a function of cognitive demands across individuals. (**c**) Estimated trajectories for individuals high (+1 SD) and low (−1 SD) on need for cognition.

We used latent growth curve modeling with bootstrapping^23^ to estimate the individual neural response in theta between 650 and 1900 ms as a function of cognitive demands (see black portion of Fig. 4a). The model showed nearly perfect fit (χ^2^ = 0.22, df = 1, p = 0.64, χ^2^/df = 0.22, CFI = 1.0, RMSEA < 0.001). We found a positive estimate for the slope (π_1_ = 0.060, p = 0.004, see thick black line in Fig. 4b), indicating that with increasing cognitive demands, participants recruited on average more cognitive resources as indexed by theta power. However, as can be seen from the ordinary least square linear regressions (grey lines in Fig. 4b), there was also considerable variability in slopes (SD = 0.076), with some individuals showing increased theta power for high, compared to low cognitive demands, whereas others show no specific response or even a reversed pattern.

### Predicting patterns of theta power by need for cognition

We investigated whether the individual slopes might serve as neural indicator of epistemic motivation by relating the slopes to need for cognition. Therefore, we administered the 18-item need for cognition scale^9^. An example item is: “I like to have the responsibility of handling a situation that requires a lot of thinking”. After adding need for cognition as a continuous variable (Fig. 4a), the model still showed good fit to the data (χ^2^ = 13.4, df = 11, p = 0.27, χ^2^/df = 1.21, CFI = 0.97, RMSEA = 0.07). We found need for cognition to significantly predict the slope of theta power in response to varying cognitive demands (λ = 0.044, p = 0.047). As illustrated in Fig. 4c, predicted values for theta power for individuals with high levels on need for cognition (SD = 1) increased with increasing cognitive demands (π_1_ = 0.104, p < 0.001), indicating that cognitive resources were dynamically allocated as required by task demands. By contrast, predicted values for theta power for individuals with low levels on need for cognition are not elevated in response to higher cognitive demands (π_1_ = 0.015, p = 0.32). With regards to our hypotheses, the overall lower theta power in the 0-back condition of individuals high, compared to low on need for cognition (r = −0.39, p = 0.01) is in line with the assumption that individuals with high levels on need for cognition invest less cognitive effort in simple tasks, compared to complex tasks, which mirrors a neuronal efficient processing^24^. Contrary, individuals with low levels on need for cognition already invest considerable cognitive resources in the 0-back condition, which would be in line with the proposed preference for simple, structured tasks^9^,^25^. Figure 2c illustrates the stronger rise in theta from 0back to 2back for individuals high compared to low in need for cognition as a time-frequency difference plot.

**Figure 5:**
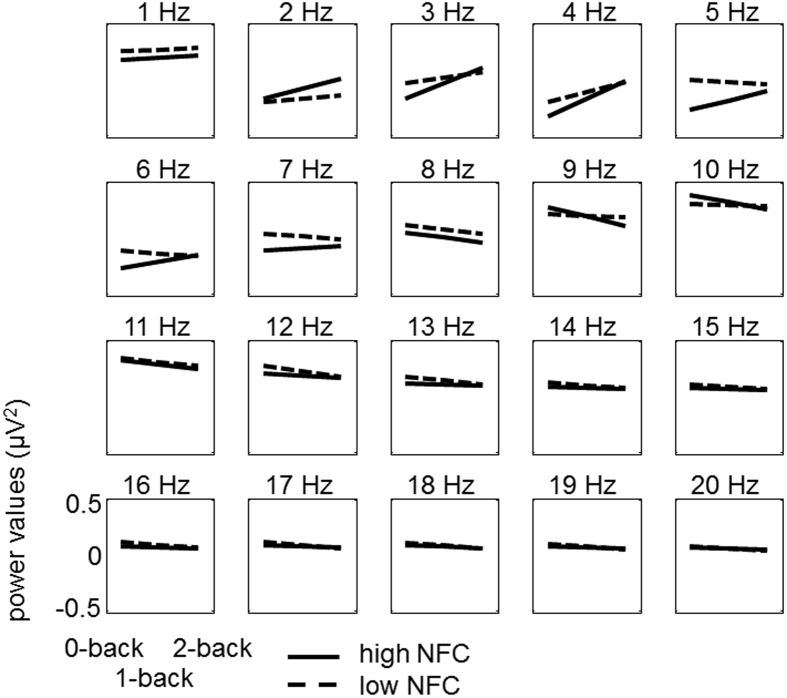
Trajectories for individuals high (solid line) vs. low (dashed line) in need for cognition, separately for 1-Hz frequency layers in the range of 1 to 20 Hz. Each figure indicates whether the power in the respective frequency layer is modulated by condition (i.e., 0-back vs. 1-back vs. 2-back).

In addition to the analyses for the 4 to 7 Hz theta band, we analyzed this effect in exploratory fashion for each frequency band between 1 and 20 Hz separately to determine the specificity to theta. Predicted slopes for individuals high (+1 SD) and low (−1 SD) in need for cognition are depicted in Fig. 5. The descriptive results suggest that the effect of a more positive slope for individuals high, compared to low in need for cognition is specific for the theta band.

### Assessing the impact of working memory capacity

As, on the one hand, epistemic motivation is moderately positively correlated with working memory capacity^26^ and, on the other, working-memory capacity has also been found to relate to theta power^11^, it is important to show that our results do indeed refer to epistemic motivation and cannot be deduced to working memory capacity alone. Therefore, we obtained a measure for working-memory capacity (see method section) for which we included an additional latent variable in the model (χ^2^ = 26.8, df = 30, p = 0.64, χ^2^/df = 0.89, CFI = 1, RMSEA < 0.001). The regression of need for cognition on the slope (estimate = 0.044, p = 0.049) was virtually unchanged when accounting for working memory capacity, while the latter had no significant influence (estimate = −0.012, p = 0.76). Therefore, the pattern of theta oscillations under varying cognitive demands as a function of need for cognition cannot be explained by working memory.

### Predicting performance

**Figure 6:**
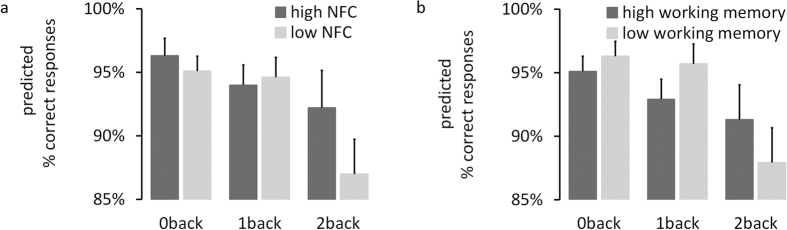
Predicted values (in percent) of correct responses in the n-back task as indicator of performance. (**a**) For scores of 1 SD above the mean and −1 SD below the mean on need for cognition (NFC), assessed by the 18-item need for cognition scale. (**b**) For scores of +1 SD above the mean and −1 SD below the mean on working memory capacity, assessed by three tasks from the Wechsler Adult Intelligence Scale. Error bars indicate 95%-confidence intervals.

Because epistemic motivation is a valid predictor of cognitive achievements^5^,^6^,^7^ and is related to individual differences in information processing^9^, we investigated whether need for cognition would also predict performance on the n-back task and, if so, whether the pattern of theta oscillations would account for the predictive validity of need for cognition. We used a mixed model approach to predict trial-by-trial performance on the n-back task. First, we found a significant interaction between need for cognition and condition on performance (F = 3.5, p = 0.03). As illustrated in Fig. 6a, individuals with low levels on need for cognition showed a stronger decrease in performance compared to individuals high in need for cognition as cognitive demands increased, with significantly lower performance levels in the 2 back-condition (p = 0.02). Second, when including working memory in the model, we found a significant interaction between working memory and condition on performance (F = 7.3, p = 0.001) with a similar pattern (see Fig. 6b); the interaction between need for cognition and condition remained virtually unchanged (F = 4.2; p = 0.02). Third, slopes of theta power significantly predicted performance, with better performance for individuals with higher, compared to lower slopes, i.e. individuals increasing cognitive resources as a function of cognitive demands (F = 7.6, p = 0.006; predicted percentage of correct responses of 95% vs. 92% for slopes +1 SD vs. −1 SD). Fourth, when accounting for slopes of theta power, we found the interaction between need for cognition and condition vanished (F = 1.6; p = 0.21), whereas the interaction between working memory and condition remained significant (F = 7.7, p < 0.001). These results suggest that the pattern of theta oscillations in response to varying levels of cognitive demands accounts for the predictive validity of need for cognition on performance, with this effect being specific to need for cognition and not more generally to other factors influencing performance such as working memory. More generally, these results are further evidence that these patterns reflect a neural mechanism underlying individual differences in epistemic motivation.

## Discussion

We identified a pattern of theta activity in response to tasks with varying cognitive demands as a neural indicator of epistemic motivation. Theta power indicated the amount of cognitive resources invested in response to situations possessing high, compared to low cognitive demands. This pattern of theta activity distinguished between individuals with high, compared to low, epistemic motivation. Specifically, latent slopes of neural activity in response to varying cognitive demands: 1) correlated with need for cognition, a personality trait reflecting stable individual differences in epistemic motivation, but not with working memory; 2) predicted performance on a cognitive task; and 3) accounted for the shared variance between need for cognition and performance on the cognitive task. Additional analyses revealed that this pattern of results was specific to theta activity, as compared to other EEG frequency bands and indicators of peripheral physiological activation.

These results add to our knowledge about functional significance of EEG theta oscillations. While there is profound evidence that power in the theta band reflects mental effort, we show that theta activity is also sensitive to stable individual differences in epistemic motivation. With regards to theories linking theta oscillations to the need for cognitive control^16^, individual differences in epistemic motivation might moderate the amount of cognitive control in response to cognitive demands, presumably due to a more efficient way in allocating their resources or differences in the associated cognitive costs. Additionally, individuals with high, compared to low levels of epistemic motivation have a higher tolerance for dealing with ambiguity and conflict^27^; therefore, the lower level of theta power in the 0back condition might indicate that these individuals perceive such situations as less conflicting^18^.

Additionally, from a personality perspective, our results provide direct evidence for the theory of the construct of need for cognition. According to the theory by Cacioppo and colleagues^9^,^25^, individuals with higher need for cognition are more prone to engage in effortful cognitive activity when given a task or making sense of the world, and are more likely to enjoy (or be less stressed by) cognitively effortful problems, life circumstances, or tasks. While the theory makes testable predictions regarding neural processes, research has mainly tested the behavioral consequences that can be deduced from the theory, such as information recall, responsiveness to argument quality, number of thoughts generated, correlation of thoughts with judgments, or subjective costs for cognitively challenging tasks^9^,^28^ or has investigated more general aspects of information processing relating to attention allocation^29^. The present study provides more direct evidence to the core assumption of the theory by obtaining an indicator of mental effort in response to a task with varying levels of cognitive demands.

Beyond the mere correlation between patterns of theta activation in response to situational demands and epistemic motivation, one may speculate about how these relations evolved. One possibility would be that individuals with high compared to low levels of epistemic motivation more often seek cognitively challenging tasks. As a consequence, higher levels of exposure and practice will lead to enhanced levels of cognitive ability, a mechanism known as environmental enrichment, and might, on a neural level, lead to a more efficient resource allocation^30^,^31^. Conversely, higher levels of exposure, practice and training might positively influence the development of epistemic motivation, as individuals who repeatedly seek exposure to cognitively challenging tasks might learn to allocate their resources in a more efficient way^32^. Finally, higher levels of cognitive ability might, on one hand, positively influence efficient resource allocation and, on the other (or as a result), increase the likelihood of successfully managing cognitively challenging situations which, as a consequence, positively influences preferences for seeking new and challenging situations, a mechanism known as environmental success^30^,^31^,^33^. Longitudinal studies with broad measures of cognitive ability are needed to clarify these mechanisms.

On a more general level, our results illustrate how personality traits can be linked to brain function. As is well known from interactionism^34^,^35^,^36^, situations vary in their relevance to any given trait and, therefore, trait differences matter to different extents in different situations. If a situation is characterized by demands that are relevant for a particular trait, they have the potential to activate this trait^37^. Individual differences on this particular trait will manifest in the type and strength of a corresponding behavioral reaction to such stimuli. We hypothesize that the neural mechanism reflecting the concept of trait activation may consist of a network functionally related to the trait domain. Situational cues that are relevant for a particular trait will thus elicit a response in this neural network. Inter-individual variability in the neural response indicates that the situational cues are processed differentially across individuals and, thus, give rise to different subsequent processing and different behavioral responses. When situational cues, corresponding neural responses to these cues, and subsequent processing and behavioral responses co-vary systematically, the activity of the particular network as a response to the situational cue provides a foundation for a particular set of responses that are manifestations of the personality trait.

The neural response to the situational cue thus constitutes what we would like to refer to as a *functional neural trait*, i.e. neural activity in response to particular cues that is moderated by individual differences on this trait. The neural response to the eliciting situational cues serves as a neural indicator reflecting individual differences on this particular trait. In line with this perspective are findings in the literature detailing neural correlates of personality that have found a moderating influence of personality on neural activity, specifically after inducing a situation that corresponds to the personality trait. For example, the neural response to wins and losses, as obtained in the feedback-related negativity^38^, has been found to be attenuated for individuals high compared to low in dispositional greed after the concept of greed was activated by situational cues^39^. Also, the relation between frontal brain asymmetry and individual differences in approach and avoidance have been found to be stronger and more robust when these traits had previously been activated^40^,^41^,^42^.

Compared to cue-invariant or static neural traits, such as resting-state electroencephalography and structural magnetic resonance imaging^43^,^44^, functional neural traits are characterized by imposing varying situational demands that are derived from a theory of the proposed construct while assessing brain-related activity that captures the proposed differences in processing. As functional traits investigate personality dispositions as processing dynamics in response to situational cues they might thus be viewed as a biological approach to the cognitive-affective personality system model^45^ and are related to and in line with the capability model of individual differences^40^,^46^. From a diagnostic perspective functional neural traits may serve as brain-based indicators of personality that have the propensity to predict and explain behavior. Such indicators may even provide a nonreactive alterative to self-report assessments that are currently the most common method for the assessment of personality, in the research context as well as in applied settings.

## Methods

### Participants

Forty-two participants were recruited from the student population of a German university. Participants were between 19 and 29 years old (on average 22.5 years, *SD* = 2.8), 36 participants were female. Participants received course credit for participation and, in addition, they were paid € 17. The study took about 2 hours. Written informed consent was obtained for all participants. For our sample size, α = 0.05 and a medium to large effect of r = 0.37, the statistical power to discover an effect if it exists in the population is (1-β) = 0.80.

### Task and Measures

The n-back task consisted of three blocks with 62 trials each. On each trial, participants saw a fixation-cross, followed by a capital letter taken from a pool of eleven letters. Each trial took 2.5 seconds (see Fig. 1). Participants were instructed to press a button with their right index finger when the letter was a target, or a second button with their left index finger if the letter was a non-target. In the first block (0-back), the target was the letter “X”. In the second block (1-back), the letter from the last trial served as target. Therefore, participants had to constantly update and memorize the target. In the third and final block (2-back), the letter presented two trials ago served as target. In addition to the 1-back condition, participants had to memorize und constantly update a second target along with the information which of the two letters was the target. Half of the trials per block were targets. The same randomized sequence of targets and non-targets and the same letters were used for all participants. Performance on the n-back task was coded trial-by-trial. In less than 1% of the trials, participants did not respond within the 2-second time span; therefore, these trials were excluded from the analyses regarding reaction time and hit rate as corresponding data were not available. Otherwise, responses were coded as correct if the correct button was pressed for either a target or a non-target, and as non-correct if the wrong button was pressed for either a target or a non-target.

For the assessment of need for cognition, the 18-item short scale was used^25^. The construct need for cognition can be located in the Big Five personality framework, pertaining to the aspect Intellect of the domain openness to experience^32^,^47^. Items were presented in German language. Participants indicated on a seven point Likert scale, ranging from “do not agree at all” to “fully agree”, the extent to which each of the statements pertained to them. Half of the items are reversed scored and were recoded before aggregating across all items. In the present study, the scale had an internal consistency of α = 0.88 (descriptive statistics were M = 84.1, SD = 14.8). For Fig. 2c, a median split was performed on need for cognition, resulting in one sub-group with low (N = 22; M = 72.7; SD = 10.3; range [39 81]) and one sub-group with high (N = 20; M = 96.6; SD = 6.7; range [84 107]) values on need for cognition.

Working memory capacity was assessed by three tasks from the Wechsler Adult Intelligence Scale^48^. The tasks were manually administered by an examiner in a one-to-one situation. Examiners were blind with regard to all other study variables (e.g., need for cognition, theta power). The first task demanded the repetition of an increasing number of digits (2 to 18); the second task was a backwards-repetition of an increasing number of digits (2 to 16). Both tasks had two items on each difficulty level. The third task consisted of the sorting and repetition of an increasing number of digits and letters (2 to 8) with three items per difficulty level; thereby, digits had to be given first, in ascending order, followed by alphabetically ordered letters. Within each task and each difficulty level, a termination criterion was defined; specifically, if all items within a difficulty level were answered incorrectly, the task was aborted. Within each task, performance was computed as the sum of all correctly answered items. Working memory performance was estimated as the aggregate of z-standardized values in the three tasks.

All methods were carried out in accordance with the approved guidelines of the Julius Maximilians University Würzburg, and all experimental protocols were approved by its ethic committee.

### EEG Recording and Quantification

While participants performed the n-back task, EEG (analog bandpass: 0.1–80 Hz, sampling rate: 250 Hz) was recorded from 31 scalp sites according to the 10–20 system, using Ag/AgCl electrodes and a BrainAmpDC amplifier (Brain Products GmbH, Gilching, Germany). Impedances were kept below 10 kΩ and electrodes were referenced to the vertex (Cz). For detection of blinks and eye-movements the vertical electrooculogram (EOG) was recorded. Data were processed offline, using Brain Vision Analyzer 2.0 software (Brain Products GmbH, Gilching, Germany). First, data were filtered, using a 0.15 Hz high-pass and a 40 Hz low-pass filter (24 dB/Octave) and, additionally, a 50 Hz notch filter. Subsequently, the EEG was segmented into epochs of 3200 ms (−800 to 2600 ms, relative to the presentation of the stimulus). Afterwards, data were corrected for ocular artifacts using an Independent Component Analysis based correction method implemented in the Brain Vision Analyzer. Larger artifacts were automatically detected by a computer algorithm implemented in Brain Vision Analyzer 2.0 software and discarded if applicable, both prior and after the ocular correction. For this purpose the following exclusion criteria were applied: (1) maximal voltage difference >250 μV within 1000 ms (prior to the ocular correction, to enhance data quality for the independent component analysis without excluding trials according to eye-related activity); (2) maximal voltage difference >100 μV within 1000 ms across the epoch and maximal voltage step of 20 μV/msec (after the correction for ocular artifacts). At least 32 artifact-free trials (on average 56 trials) were available per participant and condition. Subsequently, data were re-referenced to an averaged reference across all electrodes (excluding vertical EOG).

Event related potentials were computed by segmenting the data to −500 to 2000 ms relative to the presentation of the stimulus, averaging across all trials and correcting for baseline activity (−150 to −50 ms).

For time-frequency analyses, segmented data were convolved using a family of complex Morlet wavelets from 1 to 20 Hz in linear steps of 1 Hz. The complex Morlet wavelets are defined as Gaussian-windowed complex sine functions: 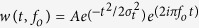, with 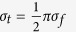 and 
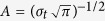
, the latter resulting in total energy of 1 for all frequency levels^49^. Constant ratio (*f*_*o*_/*σ*_*f*_) was set 6.7^50^,^51^. For each frequency layer, power values were baseline corrected by subtracting the mean activity in the time window −150 to −50 ms before stimulus onset from each data point. (Please see the supplemental material for detailed information on the rational for choosing a baseline period rather close to stimulus onset, and for results for an alternative baseline −450 to −350 ms before stimulus onset.) Segments were averaged for each participant and condition of interest. For analyses of FMθ activity, mean power values 4 to 7 Hz at Fz, F3, FCz, F4, and Cz were extracted from 650 to 1900 ms following the presentation of the stimulus and aggregated across these electrodes.

### Heart period measurement

We used three disposable Ag/AgCl electrodes (Covidien Kendall ECG Electrodes H98LG) placed according to a modified Einthoven II lead to measure heart period, defined as the time between two adjacent R-peaks^52^. The ground electrode was placed below the left collarbone, the negative electrode below the right collarbone and the positive electrode on the left side below the rib cage. The signal was digitized using a Brain Vision BrainAmp ExG amplifier (Brain Products GmbH, Gilching, Germany) with a sampling rate of 250 Hz and the BrainVision Recorder 1.20 software (Brain Products GmbH).

To analyze the event-related heart period response we first detected the peaks of the R-waves using QRSTool^53^. Following an automatic detection, the detected beats were checked and if necessary corrected manually. Next, the times of the R-peaks were exported and event-related inter-beat-intervals were extracted using a custom-built Matlab script. For every stimulus in the n-back task we extracted the inter-beat-interval surrounding the stimulus as well as the two following intervals. Heart period was defined as the interval in milliseconds between sequential R-waves and was estimated for the interval following the stimulus.

High-frequency heart period variability (HF-HPV) was estimated using the software ARTiiFACT^54^. Based on the timing of the R-peaks exported from QRSTool we computed separated inter-beat-interval series for every condition of the n-back task per participant (mean length of the series across all participants and conditions: 154.65 seconds). These inter-beat-interval series were then submitted to a frequency domain analysis of HF-HPV in ARTiiFACT. Except for the width of the Hanning window (153 seconds), standard settings of the Fast Fourier Transformation (FFT) were used (4 Hz spline interpolation, 50% overlap of resampled and detrended data). The absolute values (in ms^2^) of the HF-HPV component (0.15–0.40 Hz) were used for statistical analysis^55^. Since those values were not normally distributed, we transformed them using the natural logarithm before running the statistical analysis.

### Statistical analyses

Latent growth curve analysis^23^ with maximum likelihood function was performed using AMOS 23.0 with bootstrapping to account for the medium sample size. Latent growth curve analysis is a special case of multilevel modeling and captures the pattern of change across multiple sampling points. For each individual, a linear function described by slope and intercept was estimated. As depicted in Fig. 4a, the intercept was specified by a latent variable with regression weights fixed to 1 between the latent intercept and the three manifest variables indicating theta power in the three conditions. The slope is specified by a latent variable indicated by regression weights fixed to −2, 1, and 0 for theta power in the 0-back, 1-back, and 2-back condition, respectively. Therefore, the intercept specifies predicted theta power in the 2-back condition.

Need for cognition was modeled as latent variable indicated by three manifest variables; the latter were computed by randomly assigning each of the 18 items (see above) to one of three parcels, and subsequently averaging across these items. Working memory was modeled as a latent variable with the z-standardized values of each of the three working memory tasks as indicators.

Factor score weights were used to predict the estimated slope for each subject and each condition for subsequent analyses. We used mixed models for binomial data with a LOGIT link function for the prediction of trial-by-trial performance in the n-back task, using the GENLINMIXED procedure in SPSS 23.0.

## Additional Information

**How to cite this article**: Mussel, P. *et al*. Patterns of theta oscillation reflect the neural basis of individual differences in epistemic motivation. *Sci. Rep.* **6**, 29245; doi: 10.1038/srep29245 (2016).

## Supplementary Material

Supplementary Information
