# Supplementary material for: Patterns of theta oscillation reflect the neural basis of individual differences in epistemic motivation
Source: Sci Rep. 2016 Jul 6;6:29245. doi: 10.1038/srep29245 (PMC4933953; doi:10.1038/srep29245)
Supplement: Supplementary Information [file srep29245-s1.pdf]

Patterns of theta oscillation reflect the neural basis for individual differences in epistemic motivation

Authors: Patrick Mussel[1*], Natalie Ulrich[1], John J. B. Allen[2], Roman Osinsky[3], Johannes Hewig[1]

Supplemental material

Indicators of peripheral physiological activation. We investigated whether the pattern of responses to cognitive demands is specific for neural activity, as reflected by theta oscillation, or whether it also generalizes to peripheral physiological activation, which has also been found to be modulated by cognitive demands[1]. We obtained electrocardiographic (ECG) signals from which we identified the R-waves and computed the heart period as the time between two adjacent R-peaks, for the interval following the stimulus. Using mixed models, we found a significant main effect of condition on heart period ($F=19.7$; $p<.001$). With higher cognitive demands peripheral activity increased (i.e. shorter inter-beat-intervals: 0-back: 856 ms; 1-back: 851 ms; 2-back: 827 ms). However, need for cognition did not moderate heart rate acceleration ($F=1.3$; $p=.28$). We also investigated the spectral power in high-frequency (0.15 to 0.40 Hz) heart period as an indicator of cardiac parasympathetic activity[2]. Similarly, we found increased peripheral activity (i.e. less parasympathic activity: 0-back: 569 ms^2; 1-back: 548 ms^2; 2-back: 429 ms^2) with higher cognitive demands ($F=9.7$; $p<.001$), but no moderating influence of need for cognition on parasympathic activation ($F<1$). Therefore, as results do not generalize to ECG measure, we conclude that theta oscillations in response to cognitive demands are a unique physiological indicator for epistemic motivation.

Use of a different baseline. Due to the short pre-stimulus interval of 500ms, we chose a baseline interval [-150 -50] close to the stimulus onset to avoid that the window of the wavelet in the baseline period overlaps with the preceding trial, which would have systematically canceled out activity from the late theta period, which was our primary area of interest. However, as a baseline more distant to the stimulus onset is generally recommended[3], we repeated all analyses with a baseline of [-450 -350]ms relative to the stimulus onset and summarize the relevant results as follows:

The latent growth model positing a slope and an intercept (black part in Figure 4a) showed excellent fit ($\chi2$=0.16, df=1, p=.64, $\chi2$/df=0.16, CFI=1.0, RMSEA<.001). The estimate for the slope was positive ($\pi1$=0.031, p=.053), even though smaller in size and only marginally significant (for comparison, the original values were $\pi1$=0.060, p=.004). It can be assumed that the baseline interval more distant to the stimulus onset (i.e. [-450 -350] has removed true variance (i.e. the effect of block on theta) due to the overlapping interval of the baseline and the preceding trial.

The latent growth model including need for cognition (see Figure 4a) showed similar or slightly better model fit ($\chi2$=12.2, df=11, p=.34, $\chi2$/df=1.11, CFI=.98, RMSEA=.052). Need for cognition still significantly predict the slope of theta power in response to varying cognitive demands (λ=0.037, p=.016). Again, for the 0back-condition, there was a significant correlation between NFC and late theta (r=-.36), indicating that individuals with low levels on NFC recruit more cognitive resources in the easy 0back condition, compared to individuals with high levels of NFC.

The model additionally including working memory also shows similar fit ($\chi2$=27.2, df=30, p=.61, $\chi2$/df=0.90, CFI=1, RMSEA<.001). The regression of need for cognition on the slope (estimate=0.037, p=.016) remained significant, whereas working memory did not predict theta slopes (estimate=0.002, p=.93).

Finally, slopes of theta power significantly predicted performance (F=6.7, p=.010). When controlling for slopes of theta, the effect of NFC on performance (F=3.5, p=.03) was reduced to F=2.9, p=.052) and, thus, only marginal significant.

In sum, choosing a baseline interval more distant to the stimulus onset did not have a major impact upon the overall pattern of the results.

References

1 Gianaros, P. J., Van Der Veen, F. M. & Jennings, J. R. Regional cerebral blood flow correlates with heart period and high-frequency heart period variability during working-memory tasks: Implications for the cortical and subcortical regulation of cardiac autonomic activity. *Psychophysiology* **41**, 521-530 (2004).

2 Task Force of the European Society of Cardiology and the North American Society of Pacing and Electrophysiology. Heart rate variability: standards of measurement, physiological interpretation and clinical use. . *Circulation* **93**, 1043-1065 (1996).

3 Cohen, M. X. *Analyzing neural time series data. Theory and practice.*, (MIT Press, 2014).
